# The Assessment of Psychomotor Development in Full-Term Children at 12 Months of Age with Munich Functional Development Diagnostics Depending on the Feeding Method: A Cross-Sectional Study

**DOI:** 10.3390/pediatric15020034

**Published:** 2023-06-12

**Authors:** Grażyna Pazera, Marta Młodawska, Kamila Kukulska, Jakub Młodawski

**Affiliations:** 1 Collegium Medicum, Jan Kochanowski University in Kielce, 25-369 Kielce, Poland; 2 Clinic of Neonatology, Provincial Combined Hospital Kielce, 25-736 Kielce, Poland; 3 Esculap Student’s Scientific Society, Jan Kochanowski University in Kielce, 25-369 Kielce, Poland; 4 Department of Obstetrics and Gynaecology, Provincial Combined Hospital in Kielce, 25-736 Kielce, Poland

**Keywords:** Munich Functional Developmental Diagnosis, breastfeeding, neurodevelopment, psychomotor impairment, formula feeding

## Abstract

Background: Psychomotor development is the most important outcome determining the proper growth and development of children. Optimizing childcare and modifying risk factors can provide the child with the best conditions to realize their developmental potential. The study aimed to assess the impact of the feeding method on the psychomotor development of full-term children at 12 months of age with Munich Functional Developmental Diagnostics (MFDD). Methods: The study included 242 full-term children who were examined at 12 months of age by a child neurologist using MFDD. The children were divided into two groups depending on the feeding method: breastfed (146) vs. formula-fed (93). We analysed selected obstetric and neonatal risk factors as well as MFDD scores within the groups. Results: The only axis on the MFDD scale on which we observed a difference between the groups was social skills. No differences were noted between the groups in the analysis of the gross and fine motor skills, with regard to perception or active and passive speech. Conclusions: The full-term, exclusively breastfed infants over their first 6 months of age or longer have greater social skills in comparison with the formula-fed infants when measured on the MFDD axis.

## 1. Introduction

The child’s development is dependent on the interaction of hereditary and environmental factors. It is a complex phenomenon which encompasses somatic development, i.e., the growth of the body, and psychomotor development, i.e., the acquisition of specific skills and functions. Genetic factors determine future development, and environmental factors specify the extent to which this development is achieved. The first year of life witnesses an exceptionally intensive development of the brain. The processes of synaptogenesis and myelination of the central nervous system take place in the brain and influence the psychomotor development of the child. This process is particularly sensitive to deficiency factors. The first year of life is the so-called critical period for brain development [1]. Appropriate growth and development of children is a goal that modern medicine is constantly trying to attain. The improvement of environmental factors and reduction of risk factors, both in the intrauterine life, during delivery, and in the first months of life, is aimed at ensuring optimal conditions for the developing organism.

In the postnatal period, one of the crucial factors exerting a real impact on a growing organism is the feeding method. The feeding method which is recommended by numerous scientific societies and governmental organizations is to exclusively breastfeed for 6 months on account of short- and long-term benefits for the child and the mother, and to continue the breastfeeding, depending on the needs of the mother and the child, with the introduction of appropriate complementary solid foods at least during the first year of life or longer [2,3,4]. AAP supports breastfeeding for the first year of life or longer [2], and WHO recommends breastfeeding at least up to the child’s second birthday [3].

The best-documented benefits for a breastfed vs. formula-fed child include a positive impact on the development and functioning of the digestive and immune systems [5], as well as preventing infections [6]. Breastfeeding also brings long-term benefits in reducing the development of certain chronic diseases such as obesity [7], type 1 diabetes [8], or inflammatory bowel disease [9]. The data on the long-term effects of breastfeeding are mainly derived from cohort observational studies and may be restricted due to possible confounding factors. The above-mentioned limitations make it difficult to document the impact of breastfeeding on psychomotor development. The data so far suggest slightly better neurodevelopmental results in breastfed children in comparison with formula-fed ones, e.g., a positive effect on cognitive functions and better results in IQ tests [10]. The aim of our study was to assess the impact of the feeding method on psychomotor development in children at 12 months of age. The children were evaluated by means of Munich Functional Developmental Diagnosis (MFDD), which is one of the methods used to assess the psychomotor development of children in their first months or years of life. There are reports in the literature about the usefulness of the MFDD scale in the assessment of psychomotor development [11,12,13], mainly in the context of premature babies. However, there are no studies on full-term newborns. There is also a lack of studies using this scale in the analysis of the influence of the feeding method on psychomotor development. We hope that our study will contribute to the existing literature and establish MFDD as a practical and easy-to-use method for assessing psychomotor development in medical practice.

## 2. Materials and Methods

The study was cross-sectional. The STROBE guidelines were employed in order to properly present the report from this study [14]. The study included children who were born at the Department of Obstetrics and Gynaecology at the Provincial Polyclinical Hospital in Kielce from January 2016 to December 2018 and whose parents responded positively to the invitations sent to them. Preterm infants, children with congenital defects, children born in a severe condition (0–3 points, APGAR scale), and children exhibiting symptoms of a disease (infectious or other) at 12 months of age were excluded from the study.

The children were examined at 12 months of age by a child neurologist who had obtained a certificate to assess skills on the MFDD scale and had had over twenty years of experience. The study was conducted in the Neonatology Clinic, providing a standardized examination setting, such as an appropriately prepared room with the presence of only one related adult accompanying the child. During the study, the child had to be awake, healthy, and could not be hungry.

The study was approved by the bioethics committee at the Jan Kochanowski University in Kielce (pursuant to the bioethics committee resolution No. 14/2016). Prior to the study, the legal guardian signed an informed consent form regarding the child’s participation in the study. They also completed a questionnaire collecting information about the child, including the method of feeding the child from birth to 12 months of age.

We divided the children included in the study into two groups. In group 1 (breastfeeding), we included all those children who, until at least 6 months of age, had been exclusively breastfed. Children older than 6 months had complementary foods introduced into their diet in accordance with applicable recommendations.

In group 2 (formula feeding), we included all those children who did not meet the criteria of group 1 and were not exclusively breastfed in accordance with the current recommendations [2,3]. Therefore, group 2 included children who had only been formula-fed or received a combination of formula and mother’s milk, but in these cases, formula milk had been introduced in the first weeks/months of life. These children, after reaching 6 months of age, also had complimentary foods introduced into their diet in accordance with applicable recommendations.

The children were assessed using Munich Functional Developmental Diagnostics (MFDD). The assessment of psycho-motor development using MFDD is an accessible and easy-to-use method. The isolation of different areas covering the 8 above-mentioned functions allows us to recognize the delay in the development of each of the examined functions, which is a sufficient requirement of daily practice. This method accurately identifies children with a delay in any of the examined functions, which is important from a therapeutic point of view. It allows those infants to be selected who, due to developmental delay, require appropriate therapy. MFDD is a tool based on the standardized tables of physical development according to Hellbrugge and Pechstein [15], and it is primarily used to detect developmental delays or deficits. MFDD allows for an early diagnosis of the eight most important psychomotor functions in infancy, i.e., crawling (MFDD 1 axis), sitting (MFDD 2 axis), walking (MFDD 3 axis), grasping (MFDD 4 axis), perception (MFDD 5 axis), speaking (MFDD 6 axis), speech comprehension (MFDD 7 axis), and social skills (MFDD 8 axis). The tables in the method manual contain reference standards for a given function in each month of the child’s life. The design of the method is based on the assumption whereby each month of life is assigned certain modes of behaviour which were displayed by 90% of the examined children at that age. Therefore, the method is based on the concept of “minimal behaviour”, i.e., such behaviour that was displayed by 90% of the examined children in the respective month of life, and not on the average value or average behaviour. In this way, it was possible to assign typical behavioural patterns to a specific calendar age of the child [16]. Categorical assessment is used in MFDD, which involves determining whether a given task has been completed or not. Intermediate scores are not considered. The developmental status evaluation sheet is used to document the child’s performance in relation to specific tasks. Most of the tasks in MFDD are arranged in a way that assigns particular behaviours to a particular month of age in which they should occur. The age grades are spaced with one-month intervals. If a child has not achieved typical behavioural patterns by a certain month of life, the data are coded as negative integers corresponding to the number of months by which the child is delayed in the development of a particular function [17]. After determining the values for each area of development, we create the child’s developmental profile, which we analyse in terms of negative deviations, i.e., the researcher’s attention ought to be drawn to any downside deviation from the calendar age. A deviation of 1 month may be considered to fall within the normal range, while a deviation of 2 months always attests to a pathology [17].

The sample size was calculated to detect a minimum difference equal to at least 0.1 points on one of the scale axes at a power of 90% and an alpha level of 5%. We obtained a preliminary prevalence of differences from an earlier study [16].

The qualitative variables were presented as a percentage share of each group. The groups were compared using Pearson’s chi-squared test. In the qualitative variables, we also included the percentage share of children who exhibited a deviation in at least one axis included in one of the dimensions demarcated in the principal component analysis [16]. We presented the central tendency of qualitative variables using the mean. Standard deviation served as a measure of dispersion. In cases where a child did not achieve behavioural patterns typical for a certain age, the data values were coded as negative integers corresponding to the number of months by which the child showed a delay in the development of a particular function. We compared the groups using the Mann–Whitney U test. In the case of accompanying variables that were unbalanced across groups, we additionally performed an analysis of covariance (ANCOVA) by calculating the greatest root of Roy and its statistical significance both for the model containing all unbalanced accompanying variables and for each variable separately.

The analysis was conducted using Statistica 13.1 software (Tibco Software Inc., Palo Alto, CA, USA). We considered a difference to be statistically significant when *p* < 0.05.

## 3. Results

One thousand two-hundred invitations to take part in the study were sent. A positive response was obtained from the parents/guardians of 350 children, and of those, 242 children were included in the study. Group 1 (breastfeeding) consisted of 146 children, of whom 20 were fed mother’s milk until 6 months of age, 33 until 9 months of age, and 93 until 12 months of age. This means that 60% of full-term children who participated in the study had been fed in accordance with the applicable recommendations, i.e., they were exclusively breastfed until reaching at least 6 months of age. Out of these children, 13.7% were fed mother’s milk until 6 months of age, 22.6% until 9 months of age, and 63.7% until 12 months of age, i.e., until the start of the study.

Group 2 (formula feeding) consisted of 96 children, most of whom were introduced to formula milk right after birth or in the first quarter of their life (67.7%). Only 31 children (32.3%) were introduced to formula milk in the second quarter of their life and further feeding included exclusively formula milk.

Within the groups (breastfeeding vs. formula feeding), we analysed selected baseline demographic parameters from three periods: pregnancy, delivery, and the early postnatal period. The results are presented in Table 1. The groups initially did not differ with regard to the selected parameters, with the exception of the presence of gestational diabetes (1.37% in the breastfed group and 6.25% in the formula-fed group (*p* = 0.037)) and the mother’s education (in the breastfed group, mothers more frequently had a higher education—86.11% vs. 68.75%—than the mothers in the formula-fed group (*p* = 0.005)). Additionally, in group 1, the percentage of male children was higher compared to group 2 (60.96% vs. 46.88% (*p* = 0.031)). The birth condition of the newborns was assessed based on the percentage of children born in a moderate condition (4–7 points, APGAR scale) in the 1st and 5th minutes after birth. The clinical status of the newborns after birth did not differ between the two groups. No statistical differences were found in the analysis of weight gain and head circumference increase in the 12th month of life.

Table 2 and Table 3 present the comparison of MFDD scores for the two groups. In Table 2, the results are presented qualitatively as deviations in one of the three dimensions of the scale. The dimensions were demarcated in the principal component analysis in a separate study [16]. The dimensions consisted of the first dimension—MFDD axis 1–4, second dimension—MFDD axis 6–8, and third dimension—MFDD axis 5.

The comparison of the means of deviations on the individual axes is presented in Table 3.

Subsequently, we performed a regression analysis comparing both groups in a multiple-factor model, taking into account all the data collected in Table 1 as confounding factors. In this model, no differences in MFDD scale deviations between the groups were observed. Table 4 presents the results of the ANCOVA analysis, focusing only on the unbalanced factors between the groups, due to the extensive nature of the analysis. None of the covariates (presented in Table 1) significantly influenced the outcome of the comparison in the multiple-factor model.

The analysis of the individual functions of the MFDD scale showed statistically significant differences only in deviations of the levels of social skills (MFDD axis 8): the breastfed children did not exhibit any deviations, in contrast to the formula-fed children (0 vs. −0.0625, *p* = 0.032). We recorded no differences between the two groups in the analysis of gross and fine motor skills (MFDD 1–4), perception (MFDD 5), as well as active speech (MFDD 6) and passive speech (MFDD 7). A high percentage of children with deviations in the individual dimensions of the scale (Table 2), still falling within the developmental norm variant, may explain the assumptions of the MFDD method, in which a deviation of 1 month requires observation, but does not necessarily indicate pathology. A delay of 2 months in a specific function during the first year of life always creates suspicion of pathology [18] and requires further diagnosis.

## 4. Discussion

Our study shows that women with higher education statistically breastfeed their children more often in accordance with the current recommendations, i.e., exclusively breastfeeding for the first 6 months of a child’s life. Considering the fact that most women make decisions about how to feed before giving birth [19], it seems that education plays an important role here.

It is interesting that out of the 242 full-term babies studied, only 60% were fed only with breast milk for 6 months of life or longer in accordance with the current recommendations. These results are consistent with an analysis of the US population, where 80% of mothers start breastfeeding after giving birth, and about 60% of mothers continue to breastfeed until the end of 6 months of age [20]. The CDC report from 2022 shows that about 25% of mothers continue to exclusively breastfeed in the first 6 months of a baby’s life [21]. This indicates the need for education and cooperation in society in order to raise this percentage. 

It is also worth noting the higher, statistically significant percentage of male children in the breastfeeding group compared to the formula feeding group. The issue of the influence of the child’s sex on the method of feeding in infancy requires further research.

In our study, we assessed the psychomotor development of full-term children, born without developmental defects. Healthy newborns, born at term, who have not lost optimal development conditions due to prematurity, are not at risk of having their psychomotor development delayed. Any statistically significant difference in the achievement of psychomotor functions between the groups of the investigated children with different feeding methods seems to be of interest. This emphasizes the crucial influence of the environmental factors, i.e., the feeding method, on the psychomotor development of babies. A 2015 meta-analysis of 17 studies demonstrated that breastfeeding was associated with an improvement in IQ scores by 3.44 points in childhood and youth. This relationship was present even after a correction for the confounding factor, which is the mother’s level of intelligence [10]. The presence of long-chain polyunsaturated fatty acids in breast milk, such as arachidonic acid and docosahexaenoic acid, which promote myelination and development of the nervous system, may explain the association of breastfeeding with better psychomotor development [22]. An additional aspect that can improve the development of children is the effect of breastfeeding on establishing a closer bond between mother and child [23]. In our study, we demonstrated that in the group of breastfed babies, there were no deviations in the level of social skills (MFDD 8), and this significantly differentiated this group from the formula-fed babies (*p* = 0.032).

The eighth axis of the MFDD worksheet concerns the diagnosis of the age of social development. The child’s social development involves two closely related processes: the development of the ability to establish different relationships with other adults and children, and the gradual process of the child’s detachment, starting from the period of requiring assistance to self-reliance as a person [17]. In the diagnosis of social age in MFDD, the components of social development which reflect the infant’s reaction to the people surrounding them, i.e., the way in which the baby interacts with loved ones and strangers, were recorded. It should be noted that the skills related to the child’s acquisition of self-reliance are particularly dependent on motor development (e.g., postural motor skills, hand motor skills) and, to a large extent, on educational influences. The maturity of social behaviour is associated with appropriate psychomotor functions. In a healthy infant, the cause of social age delay generally lies in the insufficient quantity and quality of commitment on the part of a permanently present closely related person [17]. Breastfed babies are in constant and close contact with their mother, who cannot be replaced by another adult, and this is probably a factor that contributes to an exemplary score for the social skills function on the MFDD scale among 12-month-old breastfed babies. Among the formula-fed babies, deviations on this axis were recorded. There is no certainty that artificially fed children had insufficient contact with their mother, nor that among children fed with expressed milk, the level of contact was the same as in children directly breastfed. However, it is certain that breastfed children had to be in close and continuous contact with their mother. This observation requires further research. Of course, there may be other causes of social developmental disorders, such as early childhood autism or mental retardation, and only the application of developmental therapy will be able to explain whether the delay in social functioning was caused only by a deprivation syndrome or whether the baby has an intellectual disability [17]. However, we did not observe any difference between the groups in terms of motor functions (MFDD 1–4), perception (MFDD 5), as well as active (MFDD 6) and passive (MFDD 7) speech.

An analysis of the three uncorrelated dimensions of the MFDD scale determined during the PCA explaining 80.27% of the total variance (16) did not show any statistically significant differences between the groups. The loss of 20% of the variance in order to simplify the scale to three axes would not allow one to demonstrate a statistically significant difference regarding axis 8, i.e., social skills. 

Amongst the investigated children, no differences were demonstrated in physical development, including head circumference increase. There are many studies in the literature examining the influence of the method of feeding on the physical development of children. Compared to formula-fed babies, breastfed babies gain weight faster in the first 3–4 months of life and then slower thereafter. At the age of 12–23 months, there were no statistical differences in body weight between the groups [24].

The lack of group homogeneity is a limitation of our study. Such a division was the only one that could retrospectively assess babies fed only with breast milk for 6 months or more, without a supply of formula milk. Because of the size, group No. 2 had to include babies who were fed exclusively with formula or with mixed types of milk to varying degrees.

## 5. Conclusions

1. Full-term infants exclusively breastfed for the first 6 months of life or longer do not exhibit abnormalities in achieving social skills, in contrast to artificially fed infants.
2. Among full-term babies at 12 months of age, no statistically significant difference was demonstrated in terms of deviations in gross and fine motor skills, in perception as well as in active and passive speech between breastfed and formula-fed babies.
3. Women with only a primary or secondary education are less likely to exclusively breastfeed their babies up to 6 months of age.
4. Among the full-term babies investigated, 60% were exclusively breastfed up to the age of 6 months or longer in accordance with the current recommendations.

## Figures and Tables

**Table 1:** Demographic characteristics of the group.

Parameter	Group 1 (Breastfeeding)	Group 2 (Formula Feeding)	*p*
***-----Pregnancy-----***			
Singleton pregnancy	100% (146)	97.92% (2)	0.215
First pregnancy	52.74% (77)	55.21% (53)	0.386
Hypertension in pregnancy	13.01% (19)	10.42% (29)	0.542
Gestational diabetes mellitus	1.37% (2)	6.25% (8)	0.037
Hypothyroidism in pregnancy	15.07% (22)	13.54% (35)	0.741
Magnesium sulphate administration in pregnancy	0.69% (1)	0% (0)	0.881
Antenatal steroids	5.56% (8)	7.73% (7)	0.574
***-----Birth-----***			
Male sex	60.96% (89)	46.88% (45)	0.031
Nuchal cord at birth	25.34% (37)	25% (24)	0.466
Newborn’s pulmonary resuscitation	1.38% (2)	2.08% (2)	0.824
Congenital infection	0.68% (1)	1.04% (1)	0.999
Respiratory insufficiency	0.68% (1)	0%	0.416
Transfusion in neonatal period	0% (0)	0%	1.000
Apgar 1′ 4–7	6.85% (10)	5.21% (5)	0.874
Apgar 5′ 4–7	2.74% (4)	3.13% (3)	0.862
***-----Increase in 12 months-----***			
Head circumference increase over 12 months	11.40462 (1.59)	11.48353 (1.71)	0.785
Weight gain over 12 months	6451.705 (977.7)	6495.904 (1148.6)	0.712
***-----Maternal Variables-----***			
Mother with higher education	86.11%	68.75%	0.005

**Table 2:** Comparison of the percentage of deviations in individual scale dimensions between groups.

	Group 1 (Breastfeeding)	Group 2 (Formula Feeding)	*p*
MFDD 1st dimension ≠ 0	17.48%	22.92%	0.301
MFDD 2nd dimension ≠ 0	15.75%	18.75%	0.543
MFDD 3rd dimension ≠ 0	3.42%	8.33%	0.098
Any deviation in scale	17.81%	21.88%	0.434

**Table 3:** The means of deviations on the individual axes between groups.

	Group 1		Group 2		
	Mean	SD	Mean	SD	*p*
MFDD 1st dim sum	−0.447	1.336	−0.688	1.820	0.279
MFDD 2nd dim sum	−0.260	0.695	−0.458	1.479	0.519
MFDD 3rd dim sum	−0.055	0.306	−0.094	0.327	0.109
MFDD 1	−0.167	0.719	−0.198	0.776	0.893
MFDD 2	−0.027	0.233	−0.073	0.363	0.175
MFDD 3	−0.229	0.600	−0.323	0.718	0.311
MFDD 4	−0.055	0.306	−0.094	0.327	0.109
MFDD 5	−0.014	0.166	−0.052	0.420	0.340
MFDD 6	−0.240	0.602	−0.354	1.015	0.666
MFDD 7	−0.021	0.185	−0.042	0.248	0.353
MFDD 8	0	0	−0.063	0.350	0.032
MFDD sum	−0.315	0.861	−0.552	1.589	0.368

**Table 4:** ANCOVA results for models containing unbalanced covariates.

Covariate	Roy’s Greatest Root	*p*
Diabetes in pregnancy	0.119	0.212
Sex of newborn	0.113	0.248
Higher education of mother	0.138	0.133
Model with above three covariates	0.131	0.159
**for a model containing MFDD 8 as outcome**		
Diabetes in pregnancy	0.132	0.323
Sex of newborn	0.156	0.257
Higher education of mother	0.248	0.245
Model with above three covariates	0.261	0.241

## Data Availability

The data that support the findings of this study are openly available in OSF Storage at: DOI 10.17605/OSF.IO/NRQGK.
